# Evaluation design for a complex intervention program targeting loneliness in non-institutionalized elderly Dutch people

**DOI:** 10.1186/1471-2458-10-552

**Published:** 2010-09-13

**Authors:** Rianne de Vlaming, Annemien Haveman-Nies, Pieter van't Veer, Lisette CPGM de Groot

**Affiliations:** 1 GGD Gelre-IJssel (Community Health Service), P.O. Box 51, 7300 AB Apeldoorn; Academic Collaborative Centre AGORA, The Netherlands; 2 Wageningen University, Division of Human Nutrition, P.O. Box 8129, 6700 EV, Wageningen; Academic Collaborative Centre AGORA, The Netherlands

## Abstract

**Background:**

The aim of this paper is to provide the rationale for an evaluation design for a complex intervention program targeting loneliness among non-institutionalized elderly people in a Dutch community. Complex public health interventions characteristically use the combined approach of intervening on the individual and on the environmental level. It is assumed that the components of a complex intervention interact with and reinforce each other. Furthermore, implementation is highly context-specific and its impact is influenced by external factors. Although the entire community is exposed to the intervention components, each individual is exposed to different components with a different intensity.

**Methods/Design:**

A logic model of change is used to develop the evaluation design. The model describes what outcomes may logically be expected at different points in time at the individual level. In order to address the complexity of a real-life setting, the evaluation design of the loneliness intervention comprises two types of evaluation studies. The first uses a quasi-experimental pre-test post-test design to evaluate the effectiveness of the overall intervention. A control community comparable to the intervention community was selected, with baseline measurements in 2008 and follow-up measurements scheduled for 2010. This study focuses on changes in the prevalence of loneliness and in the determinants of loneliness within individuals in the general elderly population. Complementarily, the second study is designed to evaluate the individual intervention components and focuses on delivery, reach, acceptance, and short-term outcomes. Different means of project records and surveys among participants are used to collect these data.

**Discussion:**

Combining these two evaluation strategies has the potential to assess the effectiveness of the overall complex intervention and the contribution of the individual intervention components thereto.

## Background

### General Background

In the last two decades, there has been growing interest in evidence-based policymaking in the field of public health[1-4]. For this, policymakers need information about the effectiveness and cost-effectiveness of interventions to prevent disease and promote health. Public health problems do not stand alone but are embedded in macro-level socio-economic environments. Therefore, public health problems require a combination of strategies that take the local context into account[5]. As a result, there is a need for the development of appropriate evaluation designs that address these characteristics of public health interventions[5,6]. Internationally, several initiatives have been taken since the beginning of the new millennium, by bodies such as the UK Medical Research Council[5,7,8], USA Centers for Disease Control and Prevention[9] and WHO European Working Group on Health Promotion Evaluation[10], to develop guidelines for the evaluation of complex public health interventions.

In the Netherlands also, policymakers aim for more evidence-based public health interventions. For this reason, Academic Collaborative Centers for Public Health have been established since 2006[11,12]. Another step forward was the development of a national certification system for public health interventions by the National Institute of Public Health and the Environment (RIVM) in 2008. To date, only a few interventions have been approved as effective or cost-effective in the Netherlands as most evaluation studies are limited to process evaluations and therefore provide weak evidence on effectiveness[13].

The current study seeks to contribute to more evidence-based working procedures in public health practice. The aim of this paper is to provide the rationale for an evaluation design for a complex intervention targeting loneliness among non-institutionalized elderly people in a Dutch community. The intervention is practice driven, meaning that the intervention is newly developed by equitable partnering of researchers, practitioners, and policymakers directly affected by, and knowledgeable about, the local circumstances that impact health. The intervention called *Healthy Ageing *is being conducted in the community of Epe, a rural village in the eastern part of the Netherlands, with 32,970 inhabitants, 19% of whom were aged 65 years and over at the start of the initiative in January 2008[14]. The intervention commenced in September 2008 with a start package of intervention activities addressing the non-institutionalized elderly people as the primary target group and people in the social environment of the elderly as the secondary target group. The planned intervention period is two years.

Three research questions were formulated to assess the effectiveness of the complex *Healthy Ageing *project. Firstly, can we observe changes over time in the prevalence of loneliness and in the determinants of loneliness in the general non-institutionalized elderly population of the intervention community, Epe, and specifically in high risk groups? Secondly, can these changes be attributed to the complex intervention? Thirdly, how can the observed changes be explained and what are the active components of the intervention?

For the purpose of this paper, the term *complex intervention *is defined as an intervention consisting of several *interacting components*[8]. The *components *may include actions and activities at the individual level and at the social and physical environmental level. The *level of complexity *may be influenced by the number of components, the interactions between components, the number and difficulty of behaviors required by those delivering or receiving the interventions, the number of groups or organization levels targeted by the intervention, the number and variability of outcomes, and the permitted degree of flexibility or tailoring of the intervention[8]. This complexity makes a classical randomized controlled trial (RCT) design - generally accepted as the gold standard design for evaluating the efficacy of bio-medical trials in a clinical or controlled setting - inappropriate for evaluating the effectiveness of public health interventions in a real-life setting[15,16]. Restricting the success indicator to one single health or behavioral outcome leads to many unsolved questions about the success factors for, and barriers to, the effectiveness of the intervention[6,17]. Therefore, an evaluation approach is proposed that includes a combination of quantitative and qualitative evaluation methods to answer the three research questions of this study. To answer the first and second question, a quasi-experimental pre-test post-test study design including short-term, mid-term and long-term outcome indicators is used. To be able to answer the third question, intervention inputs, activities, and outputs are recorded to assess the implementation process. In-depth qualitative research is used to investigate the acceptability of the project within the target population in more detail.

### Background to Healthy Ageing

**Figure 1:**
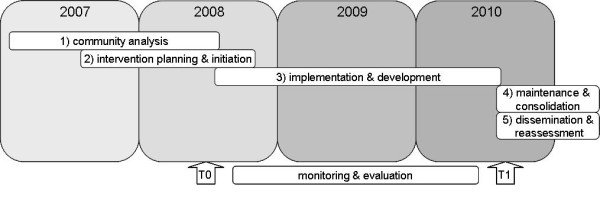
**Timeline intervention and evaluation planning**.

Local policymakers in Epe defined loneliness as one of their priority areas, as local data showed that 40% of the elderly were mildly to severely lonely[18]. To develop an intervention program, a project group was commissioned, including representatives of the municipality of Epe, the regional community health service, the regional mental health service, and the local welfare organization for the elderly. The activities of the project group are described according to first two phases of Bracht et al.'s[19] community organization model: the community analysis phase and the intervention planning and initiation phase. The remaining three phases, the implementation phase, the maintenance and consolidation phase, and the dissemination and reassessment phase are beyond the scope of this paper. In Figure 1 the different phases of the project are indicated on a timeline. However, it should be borne in mind that the succession from one phase to another is not clear cut.

The first phase comprises the community analysis, also called context analysis or needs assessment, in combination with a literature study to identify the causes of loneliness and potential solutions to prevent or diminish loneliness. The community analysis includes in-depth analysis of local monitoring data and interviews with the elderly, organizations, and policymakers to discover the most important risk factors for loneliness in the local population and to generate ideas for an intervention strategy.

In the literature, loneliness is described as a discrepancy between the desired and realized social contacts of an individual [20]. This negative experience may be related to the absence of a partner or close relative, called emotional loneliness, or due to minimal social integration and the absence of friends with common interests, described as social loneliness. As the causes of loneliness are very diverse, different approaches are needed for different subgroups. Three potential pathways to reduce feelings of loneliness can be distinguished, namely network development, lowering of standards, and adjusting the relevance of the experienced loneliness [21]. Network development concerns an interaction between an individual and his or her social environment. The other two solution pathways require more intrinsic changes.

The local monitoring data show that elderly people have a higher risk of becoming lonely if they have physical limitations, have difficulty managing on their income, are recently widowed, or have mental disabilities. On the other hand, frequent involvement in social engagement activities appears to be related to better self-perceived health, better mental health, and better physical functioning. Furthermore, higher contact frequency and better appreciation of contacts with friends, family, and neighbors are related to better health. Remarkably, satisfaction about contacts with neighbors is most strongly related to health[22]. The important role of neighbors is confirmed by the interviews with the target population. In addition, these interviews show that elderly citizens experience their health and wellbeing in the context of their daily life and not as isolated issues. They may benefit most from a positive approach, the provision of services in the immediate neighborhood, improved information provision about these services, and cooperation between service providers in the community [23].

The second phase in intervention planning is the design and initiation phase. In this phase, the project group formulates the overall project aim. The project aim is to reduce loneliness among non-institutionalized elderly people aged 65 years or over by 10% in two years, i.e. from a mean score of 2.6 to 2.4 on the loneliness scale of De Jong-Gierveld. For the purpose of the evaluation design as described in this paper, the most important sub-objectives are: (1) to reduce loneliness in the high risk groups (physical limitations, low income, recent widowhood, mild mental disabilities); and (2) to create more awareness about the existence of loneliness in the general population.

An overview of the intervention activities addressing different target groups is given in Additional file 1. Intervention activities for the high risk groups are directed at the development of a stronger personal network and skill training (objective 1). These activities include psychosocial courses based on the principles of life history memory [24-26], and social activities organized by the local welfare organization. The general elderly population is being approached by means of a mass medial campaign including a monthly article in the local newspaper, distribution of posters, and information meetings. This campaign aims to increase the awareness of the prevalence of loneliness among elderly people (objective 2), to give general lifestyle advice to improve healthy ageing, and to provide information about how to support each other with emotional or practical problems. As loneliness is not an isolated problem, the local newspaper articles are also directed at the social environment of the elderly, e.g. their family and other relatives, from now on called 'general Epe population', professionals and volunteers working with elderly people, and policymakers. Furthermore, professionals and volunteers are being trained to recognize early symptoms of loneliness and to make their diagnosis a subject of discussion. Moreover, these intermediaries are informed about the intervention activities and each other's services by a newsletter distributed three times a year.

The intervention activities introduced in the first project year have continued in the second year. Furthermore, initiatives of citizens to organize social activities are being stimulated within the intervention component *Neighbors Connected*. Simultaneously, the local government is being supported in the development of their new policy document in order to ensure that newly developed initiatives are embedded in the regular activities of public health practitioners.

## Methods/Design

### Logic Model for Loneliness Prevention

**Figure 2:**
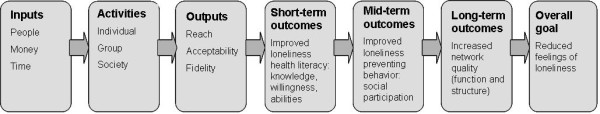
**Logic model for loneliness prevention at the individual level**.

A logic model has been developed to guide the evaluation planning (Figure 2). The model focuses on the causal chain between intervention activities and outcomes at the level of the primary target group. In this model, reduction of the prevalence of loneliness is placed as the overall goal. *Improvement of the network quality *is defined as an early marker for loneliness reduction and the long-term outcome of the intervention. Network quality is defined as a combination of the structure and function of the network. Therefore, improvement of network structure and improvement in experienced social support (network function) have been chosen as indicators for network quality. Improvement of the behavioral outcomes *being socially engaged *and *searching for professional or informal aid to support social engagement if needed *are included as mid-term outcomes. Thereafter, improvement of knowledge, attitude, and abilities are formulated as short-term outcomes, according to theoretical behavioral models [27]. These constructs are defined as *loneliness health literacy *in the model and will be achieved if sufficient outputs are delivered in terms of reach, dose received, and acceptability. Based on this model, appropriate indicators and research methods have been selected to measure these outcomes. These indicators are described in the section *Questionnaire Development *and in Additional file 2. The model serves to guide the evaluation both of the overall complex intervention and of the individual intervention components.

### Evaluation Design for Healthy Ageing

In this section, the research approach to evaluate the overall effect of the complex intervention is described, building on a quasi-experimental pre-test post-test design involving a control group. By so doing, research questions 1) Can we observe changes over time in the prevalence of loneliness and in the determinants of loneliness? and 2) Can these changes be attributed to the complex intervention? are addressed.

As already stated, Figure 1 visualizes the evaluation activities on a time line. A control community comparable to the intervention community was selected on the basis of demographic characteristics such as number of inhabitants, proportion of elderly persons in the community, religious orientation, and urbanization grade. Adjacent communities were excluded from consideration as controls in view of the potential contamination of the project activities. Table 1 indicates that the populations of the intervention and control community are comparable in terms of demographic characteristics, determinants of loneliness, and prevalence of loneliness at baseline. In the control community as well as in the intervention community, regular health care, social activities, and other services are provided by, e.g., the community health service, local welfare organizations, home care organizations, housing agencies, and volunteer organizations. Local policymakers in the control community have been asked to restrict the starting of new initiatives for the elderly during the study period.

**Table 1:** Baseline characteristics (%)^1 ^of intervention and control communities

		Intervention (n = 905)	Control (n = 897)
**Gender**	Men	44	43
**Age**	65-75	52	50
	75>	49	50
**Marital status**	Married	67	65
	Unmarried	3	4
	Divorced	3	4
	Widowed	27	28
**Education level**	Non/primary	24	22
	Lower	47	44
	Intermediate	13	17
	High	16	18
**Managing on income**	Difficulties	12	9
**Country of birth**	Netherlands	97	97
**Household composition**	1-person	30	34
**Living situation**	Fully independent	93	92
	With services	7	9
**Loneliness**	Not lonely	50	52
	Mildly lonely	41	41
	Severely lonely	7	5
	Very severely lonely	3	2
**Self-perceived health**	Good to excellent	73	76
	Moderate to bad	28	25
**Functional status**^**2**^	Not disabled	62	63
	Disabled in IADL	18	16
	Disabled in MADL/IADL	14	15
	Disabled in all domains	7	7
**Mental health**	Good	83	88
	Mild problems	13	9
	Moderate problems	4	2
	Severe problems	1	1

1 Due to rounding off percentages may exceed 100%

2 Domains of functional status: basic activities of daily life (BADL), mobility activities of daily life (MADL), instrumental activities of daily life (IADL)

#### Study Sample

The sample size calculation is based on an estimated reduction in loneliness of 10% at the population level. This means that a 10% difference in the mean score for loneliness on the loneliness scale of De Jong-Gierveld between the intervention and control community has to be detectable (α = 0.05;1-β = 0.80). Standard deviation of difference in loneliness was estimated as SD = 2.0 based on experiences in the Longitudinal Ageing Study Amsterdam (personal communication Prof. Van Tilburg). This leads to an effect size of d = 0.13. The calculated sample size (n = 930) was raised to 1,350 because of an expected response rate of 70%, based on previous experiences of the community health service in local surveillance studies among elderly people.

A random study sample of non-institutionalized people aged 65 years and over was selected from the municipal registration system in both the intervention and the control community. People aged 75 years or over were oversampled to constitute half of the study population.

#### Data Collection

Baseline measurements were taken over an 11-week period from mid August 2008 to the end of October 2008. The follow-up measurement is scheduled to take place in the same period in 2010. Baseline data were collected by means of a 20-page, 60-item, self-administered questionnaire. Potential participants received an information letter together with the questionnaire at their home address. In this letter, it was explained that agreement to participate in the study was confirmed by the elderly person returning the questionnaire. A central telephone number was provided for questions concerning the study or to ask for assistance with filling out the questionnaire. In addition, the participants were allowed to ask a relative for assistance. Two reminders were sent out four and seven weeks after the first letter. The second reminder included another copy of the questionnaire. The response rate was 50% after four weeks, 58% after six weeks, 72% after nine weeks, and 74% when the baseline study closed after 11 weeks. Blank questionnaires were removed. This resulted in a study sample of 905 participants in the intervention community and 897 participants in the control community, respectively; this corresponds with a response rate of 67%.

The study is not invasive to the study participant's integrity. Therefore it does not require formal ethics review according to the criteria of the Medical Research Involving Human Subjects Act. The use of personal data in this study is in compliance with the Dutch Personal Data Protection Act and the Municipal Database Act, and has been registered with the Dutch Data Protection Authority (number1440826).

#### Questionnaire Development

Inclusion of the indicators for determinants of loneliness in the questionnaire is based on the logic model for loneliness prevention (Figure 2). An overview of these indicators is given in Additional file 2. In addition to the determinants of loneliness, demographic, lifestyle, and health indicators are included in order to characterize groups at risk. The indicators have been mainly selected from the standards of the national surveillance system for adults and the elderly in the Netherlands[28]. These national standards are based on best available scientific insights, experiences of local community health services, and expert opinions. For the indicators not included in the national surveillance system, the international scientific literature was reviewed. The questionnaire was pre-tested in a group of five voluntary elderly advisors to assess social acceptability of the questions by the local population and applicability for self-administration. Thereafter, the questionnaire was slightly adapted.

#### Exposure Assessment

In theory, all elderly people in the intervention community are more or less extensively exposed to the intervention components and people in the control community are not. However, in order to be able to explain the observed success or failure of the intervention in terms of changes in the prevalence of loneliness and in the determinants of loneliness and to contribute to research question 3, it is important to gather information about the true exposure (also called dose received) of individual elderly persons from the intervention community within the study sample. Therefore, during the follow-up measurement study towards the end of 2010, participants will be asked whether they have read something about the intervention in the local newspapers, heard about the intervention in another way, have participated in one of the courses or have attended an information meeting.

### *Evaluation of Individual Components of *Healthy Ageing

Complementary to the effect evaluation of the overall complex intervention, the individual intervention components have to be evaluated. This part of the evaluation delivers information to answer research question 3) How can the observed changes be explained and what are the active components of the intervention? Additional file 3 gives an overview of the intended evaluation activities. Evaluation of the inputs, activities, and outputs of the intervention are part of the process evaluation and include indicators for dose delivered, integrity, reach, dose received, and acceptability. Furthermore, the effect evaluation of the individual intervention components focuses on what has been achieved in the short term in terms of changes in behavioral determinants, behavioral intentions, and perceived further benefits. As the intervention is ongoing and dynamic, the evaluation activities take place throughout the life of the program. In addition, in-depth qualitative research will be conducted to understand the acceptability of the intervention activities to the target population.

#### Inputs

Project group members record all their personal inputs in the project, such as time investment, allocated resources, costs, organizational issues, and contact administration. In this way, it becomes clear which factors are needed to develop a well-functioning project group capable of coordinating, preparing, and organizing intervention activities. Furthermore, minutes of meetings are used to study the decision-making processes. The Checklist of Coordinated Action[29] was used at the end of year one to evaluate the experiences of the project group members and their managers about the collaboration and will again be used at the end of the intervention period to make the final evaluation.

#### Activities - Dose Delivered

To assess the dose delivered, the project group members record the actual delivery of intervention activities, such as the number of articles published and the number of courses and meetings organized. All this information is collected in a database. In the database, some characteristics of every intervention activity are also recorded, such as the general objective of the activity, the intended target group, a general description of the content of the activity, the type of activity (e.g. information and education, community development, or policy development), the level of participation of the target group, the setting, the duration of an activity (e.g. once-off or repeated meetings), the length of meetings, and the interval between meetings. Data collected about inputs and activities contain information about the integrity of the program, i.e. whether the program is being implemented as planned.

#### Outputs - Reach, Dose Received, Acceptability

The reach of the intervention is assessed by counting the number of participants per activity. Participants' general characteristics, i.e. gender, age, and occasionally indicators to recognize high risk groups, namely marital status, functional status, mental status, and loneliness are estimated by the course leaders or if possible reported by the participants on an evaluation form.

The actual dose received by elderly people in the intervention community is assessed by different means. During the courses, frequency of attendance is recorded for each participant. This is a measure of *dose per activity*. However, participants on these courses are not per definition included in the sample of the pre- and post-test. Therefore, complementary to registration of *dose per activity*, *dose per individual *is assessed among study participants of the pre- and post-test. They will be asked in the follow-up measurement about their involvement in the intervention activities as described in the section, *Exposure Assessment*.

At the end of each intervention activity, apart from the communication materials (posters and flyers), the participants are asked to rate how they valued the activity. The questions are linked to the content of the activity, and the information collection methods vary from informal feedback to one-page evaluation questionnaires in the form of a visitors' book and the longer traditional evaluation forms. Two other qualitative in-depth studies have been designed to gain more insight into the motivations for participation in the intervention activities and the value derived from them. In the first study, *Neighbors Connected *is evaluated using in-depth interviews with elderly people who organize or participate in an activity[30]. The second qualitative study will be conducted among a sample of less active elderly people in the community to assess their opinion concerning communications about different intervention activities, the barriers they experience to participating in an activity, the factors that make an intervention attractive, and their perceived benefit of participating in one of the activities.

#### Short-Term Outcomes

Short-term outcomes at the individual level comprise the behavioral determinants. Using a short evaluation form or via informal feedback after the information meetings and courses, the participants are asked what they have learned. Participants in the psychosocial courses are asked whether their discomforts diminished after the course and whether they perceived an increase in knowledge and skills. Contact details of participants are collected after the intervention activities to have the opportunity to assess the effects of the activities after some months. In this follow-up, questions about changes in attitude and behavior are asked.

## Discussion

The evaluation design as presented in this paper sets a framework for the evaluation of the complex intervention *Healthy Ageing *and aims to contribute to more evidence-based working procedures in public health practice. Combining two research strategies, namely the evaluation of the overall complex intervention and the evaluation of the individual intervention components provides, in our opinion, a promising way to evaluate complex public health interventions. First, a range of outcome indicators is included to assess short- and long-term outcomes. Second, different measures are used to assess the exposure of the target population to the intervention components. Third, in-depth qualitative research is conducted at the end of the research period to access the acceptability of the intervention by the target population.

Evaluation of a complex intervention conducted in a real-life setting has implications for the design. The *Healthy Ageing *project is a practice-driven intervention; this means that the intervention activities have been developed in cooperation with local public health practitioners and policymakers. As a consequence, the intervention is not fixed from the start. Intervention activities may be adapted and room is provided for local initiatives and activities. This working procedure requires a flexible attitude on the part of researchers, and the evaluation design has to be sensitive to consider the on-going development of the project.

Moreover, the *Healthy Ageing *project is a complex intervention including a combination of intervention components that reinforce each other and interact with the local context. As a consequence, the exposure is not under the full control of the project group. Therefore, the intervention dose received by the target group is expected to differ between individuals. Related to this, the expected progression from short-term to mid-term and long-term outcomes depends on the dose of the intervention. Therefore a whole range of outcome measures has to be included in the data collection.

Given these characteristics of complex interventions, a combined evaluation strategy, including qualitative and quantitative research methods to assess outcome indictors over the entire logic model, has been chosen to assess the effectiveness of the complex intervention and to understand the underlying processes. To answer research questions 1) Can we observe changes over time in the prevalence of loneliness and in the determinants of loneliness? and 2) Can these changes be attributed to the complex intervention? it will be important to consider the robustness of the design and the choice of exposure and outcome measures[5].

With regard to robustness, a quasi-experimental pre-test post-test study design has been chosen as an alternative to an RCT to measure changes in loneliness and determinants of loneliness. Randomization of either individuals or communities to the intervention or control group was not desirable as the *Healthy Ageing *project was initiated in a local community that was motivated to promote the health and wellbeing of its elder citizens. It proved possible to select a control community comparable to the intervention community in terms of demographic characteristics, health status, and the main determinant of interest, namely loneliness and determinants of loneliness. Adjacent communities were excluded from consideration as possible controls to prevent diffusion from the intervention to the control group. Participants in the intervention and control community were randomly selected from the municipal registries and can be considered as representative of the non-institutionalized elderly population. The presence of a control group makes it possible to measure the effect of the intervention by making adjustments for confounding factors that may influence loneliness.

Sample size is another important component influencing the robustness of the design. The study population should be large enough to account for variability in individual-level outcomes. Therefore a power calculation was made to calculate the necessary study size, sensitive enough to detect a 10% reduction in loneliness. Although the response rate was reasonably high (67%) in both the intervention and control community, during the baseline measurement it was below the intended 70%. A high response in the follow-up measurement will be necessary to ensure sufficient power and to enable subgroup analyses for the high risk groups.

Finally, the condition of standardization of the exposure within an RCT is contravened in a complex intervention in a real-life setting. In the case of the *Health Ageing *project, no protocols have yet been developed to enable the implementation of the intervention in a standardized way. However, even if there were protocols available, these would have to be tailored to the local context. Nevertheless, this limitation will be overcome by the assessment of a range of exposure measures, including inputs in terms of time, manpower and resources, the dose delivered, reach, and dose received by the target population.

The choice of outcome measures is based on the logic model. The literature and in-depth analysis of local monitoring data prompted the selection of indicators for network structure and network function, social engagement, and health literacy. Changes in these indicators can be seen as intermediate outcomes for the reduction of loneliness or as mediator between intervention and final outcome.

The third research question concerns the explanation of the observed effects and analysis of the active components of the intervention. This information will be essential to make the project transferable to other communities.

The evaluation of individual intervention components in this study aims to discover facilitating and inhibiting factors along the causal chain of the logic model. These factors can be attributed to the delivery of the intervention by the project group, or the acceptance of the intervention by the target population.

Accordingly, to move from inputs to activities, the contribution of the project group members in terms of time, resources, and expertise has to be assessed. These are preconditions for the implementation of the planned intervention activities. Thereafter, to move from activities to outputs, project group members record the actual activities undertaken and the number of participants reached. During regular meetings, difficulties faced and successes achieved are discussed in more detail. The next step is to move from outputs to short-term outcomes. This step is evaluated in two different ways. First, participants in courses and information meetings are asked about their appreciation of the activity and about the acquired skills or knowledge. Second, in-depth qualitative studies provide insight into the motivations of the target population to attend - or not to attend - certain intervention activities. Furthermore, insight into perceived usefulness and outcome expectation are of interest because these factors may stimulate elderly people to participate, or discourage them from participating.

To conclude, combining two research strategies, namely the evaluation of the overall complex intervention and the evaluation of the individual intervention components, has in our opinion the potential to answer our three central research questions. The pre-test post-test study design delivers information about changes over time in the prevalence of loneliness and in the determinants of loneliness in the general elderly population. The presence of a control community makes it possible to exclude the influence of confounding factors from these observations. Complementarily, the evaluation of the individual intervention components provides information about the implementation process. These data explain how the objectives are achieved or not, and contribute to improvement of active components. Altogether, the collection of essential information to transfer the project to other communities is assured.

## Competing interests

The authors declare that they have no competing interests.

## Authors' contributions

RV, AH, PV, LG contributed to the design of the study and the development of the manuscript. All authors provided comments, read, and approved the final manuscript.

## Authors' information

All authors are connected with the Academic Collaborative Centre AGORA.

RV is a PhD candidate at Wageningen University, Division of Human Nutrition, Chair Nutrition and Epidemiology. She is employed as an epidemiologist at the community health service (GGD Gelre-IJssel). She is a researcher in the project group in Epe with the main responsibility for evaluating the project.

LG (1^st ^promoter) is professor of Nutritional Physiology focusing particularly on the ageing process and the aged, Division of Human Nutrition, Wageningen University. She is involved as project leader in AGORA, expert in nutrition for the elderly.

PV (2^nd ^promoter) is professor of Nutrition and Epidemiology, Division of Human Nutrition, Wageningen University. He is chair of the Academic Collaborative Centre AGORA, expert in epidemiologic methodology.

AH (co-promoter) is assistant professor at Wageningen University, Division of Human Nutrition, Chair Nutrition and Epidemiology and epidemiologist at the community health service (GGD Gelre-IJssel). She is the coordinator of AGORA.

## Pre-publication history

The pre-publication history for this paper can be accessed here:

http://www.biomedcentral.com/1471-2458/10/552/prepub

## Supplementary Material

Additional file 1 **Overview of intervention activities, the target groups, and intended objectives within *Healthy Ageing***. Schematic overview of the individual intervention components e.g. press releases, newspaper articles, posters, flyers, information meetings, courses, social activities, *Neighbors Connected*, Newsletter, workshop, round table discussions and lobby work. For each activity the intended target population, a description of the content and the objective of the activity is given. Click here for file

Additional file 2 **Indicators included in questionnaire pre-test post-test**. Schematic overview of the key-indicators included in the questionnaire of the pre-test and post-test. For each indicator it is explained which concepts are measured. Besides, the number of items and scale characteristics are given. Click here for file

Additional file 3 **Indicators and methods to assess inputs, activities, outputs, and outcomes within *Healthy Ageing***. overview of the research activities in the process and effect evaluation of *Healthy Ageing*. Research activities are ordered along the components of the *Logic Model for Loneliness Prevention *(input, activities, outputs, short-term outcomes, mid-term outcomes, and long-term outcomes). Per intervention component the indicators and data collection methods are given. Click here for file
