# Supplementary material for: Evaluation design for a complex intervention program targeting loneliness in non-institutionalized elderly Dutch people
Source: BMC Public Health. 2010 Sep 13;10:552. doi: 10.1186/1471-2458-10-552 (PMC2945949; doi:10.1186/1471-2458-10-552)
Supplement: Additional file 1 — Overview of intervention activities, the target groups, and intended objectives within Healthy Ageing. Schematic overview of the individual intervention components e.g. press releases, newspaper articles, posters, flyers, information meetings, courses, social activities, Neighbors Connected, Newsletter, workshop, round table discussions and lobby work. For each activity the intended target population, a description of the content and the objective of the activity is given. [file 1471-2458-10-552-S1.DOC]

**Additional files**

Evaluation design for a complex intervention program targeting loneliness in non-institutionalized elderly Dutch people

**Authors**

Rianne de Vlaming^1*^, Annemien Haveman-Nies^1, 2^, Pieter van ’t Veer^2^, Lisette CPGM de Groot^2^

^1^ GGD Gelre-IJssel (Community Health Service), P.O. Box 51, 7300 AB Apeldoorn, the Netherlands; Academic Collaborative Centre AGORA

^2^ Wageningen University, Division of Human Nutrition, P.O. Box 8129, 6700 EV, Wageningen, the Netherlands; Academic Collaborative Centre AGORA

**Additional file 1: Overview of intervention activities, the target groups, and intended objectives within *Healthy Ageing***

| **Activity** | **Target groups** | **Description** | **Objectives** |
| --- | --- | --- | --- |
| **Press releases and free publicity** | General population  General elderly population  Intermediaries | Several press releases are disseminated through local media | To create awareness about intervention activities directed at healthy ageing |
| **Articles in newspaper** | General population  General elderly population | Monthly information article in local newspaper about different topics, e.g. bereavement, coping with physical limitations, optimism, participating in social activities | To increase awareness about personal opportunities to maintain health and quality of life  To increase awareness about the opportunities in Epe to be involved in social activities  To increase awareness about the care and welfare facilities in Epe |
| **Posters**  ***Healthy Ageing*** | General population  General elderly population | Poster with one-liner relating to healthy ageing, disseminated among intermediaries and in public places | To increase awareness about the importance of social and emotional wellbeing |
| **Posters**  ***Neighbors Connected*** | Active elderly people | Poster with information about *Neighbors Connected*, disseminated among intermediaries and in public places | To increase awareness about the possibility to get financial and organizational support in organizing an activity in the neighborhood |
| **Flyers**  ***Neighbors Connected*** | Active elderly people | Flyer with information about *Neighbors Connected*, disseminated via intermediaries, distributed among the elderly with personal explanation | To increase awareness about the possibility of getting financial and organizational support in organizing an activity in the neighborhood |
| **Information meetings** | General elderly population | Interactive presentation with 10 tips about healthy ageing hosted by organizations for the elderly | To increase awareness about personal opportunities to maintain health and quality of life  To increase awareness about the opportunities in Epe to be involved in social activities  To increase awareness about the opportunities for professional help with personal problems |

**Additional file 1: Overview of intervention activities, the target groups, and intended objectives within *Healthy Ageing* (Continued)**

| **Psychosocial course**  **‘Look for a meaningful life’** | Elderly with mild depressive symptoms | Course based on principles of reminiscence delivered by the mental health service | To increase social communication skills  To stimulate the experience of a positive self-image, more self-efficacy, a meaningful life, a better quality of life, and diminished feelings of gloom |
| --- | --- | --- | --- |
| **Psychosocial course**  **‘Life stories’** | Elderly with mild depressive symptoms | Course based on principles of reminiscence delivered by the mental health services | To increase social communication skills  To stimulate the experience of a positive self-image, more self-efficacy, a meaningful life, a better quality of life, and diminished feelings of gloom |
| **Psychosocial course**  **‘Living with a chronic disease’** | Elderly with a chronic disease | Course based on principles of reminiscence aimed at coping with physical limitations | To increase awareness about the causes of stress and variations in mood  To increase skills to cope with limited energy  To stimulate the experience of more self-efficacy, a better quality of life, and diminished feelings of gloom |
| **Social activities** | General elderly population | Diverse activities organized by the welfare organization for the elderly | To increase social engagement of the elderly and strengthen their social network |
| **Activities of *Neighbors Connected*** | Less active elderly | Diverse activities initiated by *Neighbors Connected* | To increase social engagement of the elderly and strengthen their social network  To increase the three dimensions of Sense of Coherence: meaningfulness, manageability, and comprehensibility |
| **Newsletter** | Intermediaries | Newsletter with information about different topics concerning healthy ageing | To inform intermediaries about activities of the project group  To inform intermediaries about each other’s expertise and services |

**Additional file 1: Overview of intervention activities, the target groups, and intended objectives within *Healthy Ageing* (Continued)**

| **Workshops to recognize symptoms of loneliness** | Intermediaries | Half-day training about how to recognize early symptoms of loneliness and how to make this observation a subject for discussion | To increase awareness about the common prevalence of loneliness among the elderly  To increase knowledge and skills to recognize early symptoms of loneliness  To increase skills to speak about observed symptoms of loneliness with clients  To increase awareness about services of other professionals |
| --- | --- | --- | --- |
| **Round table discussions** | Intermediaries  Policymakers | Yearly meeting with stakeholders | To stimulate cooperation between intermediaries  To develop a prevention network around the elderly |
| **Lobby work** | Intermediaries  Policymakers | Individual meetings with intermediaries and policymakers | To stimulate cooperation between intermediaries  To develop a prevention network around the elderly |
