# Supplementary material for: Evaluation design for a complex intervention program targeting loneliness in non-institutionalized elderly Dutch people
Source: BMC Public Health. 2010 Sep 13;10:552. doi: 10.1186/1471-2458-10-552 (PMC2945949; doi:10.1186/1471-2458-10-552)
Supplement: Additional file 2 — Indicators included in questionnaire pre-test post-test. Schematic overview of the key-indicators included in the questionnaire of the pre-test and post-test. For each indicator it is explained which concepts are measured. Besides, the number of items and scale characteristics are given. [file 1471-2458-10-552-S2.DOC]

**Additional file** 2: Indicators included in questionnaire pre-test and post-test

|  | **Indicator** | **Description** | **# items** | **Scale** |
| --- | --- | --- | --- | --- |
| **Loneliness** | Loneliness scale of De Jong-Gierveld [1, 2] | Scale consists of 6 positively formulated statements indicating emotional loneliness and 5 negatively formulated statements indicating social loneliness | 11 | 3-point scale:  yes; more or less; no |
| **Determinants of loneliness** | | | | |
| **Social participation** | Social activities of daily life [3] | Scale consists of 8 domains of social participation: doing paid work, doing voluntary work, delivery of informal support, membership of a society, participating in cultural activities, participating in recreational activities, regular maintenance of social contacts, passive engagement | 26 | 4-point scale with extremes from (almost) daily to (almost) never |
| **Network structure** | Network typology of Wenger[4] | Questions about geographical distance from close relatives, contact frequency, and attending church or societies  Five network types are identified: family dependent, locally integrated, locally self-contained, wider community focused, private restricted. | 8 | Geographical distance: within same household, 1.5 km, 1.5-8 km, 9-24 km, 25-80 km, more than 80 km, n.a.  Frequency: daily, 2-3 times a week, at least weekly, at least monthly, never, n.a.  Social involvement: regularly, occasionally, never |
| **Network function** | Social Support List (SSL-12)[5] | Scale consists of 3 domains: everyday support, support in problem situations, esteem support  Statements concern experiences in regular social contacts | 12 | 4-point scale with extremes from never to very often |
| **Health indicators** | | | | |
| **Functional status** | Hierarchical abilities of daily living (ADL)[6] | Scale consists of 3 domains: basic activities of daily life (BADL), mobility activities of daily life (MADL), instrumental activities of daily life (IADL) | 13 | 3-point scale: without difficulty, with difficulty, only with assistance |
| **Self-perceived health** | Self-perceived health | Direct question: How would you describe your health in general? | 1 | 5-point scale with extremes from excellent to very bad |
| **Mental health** | Mental Health Inventory (MHI-5)  [7, 8] | Feelings of wellbeing in the previous month | 5 | 6-point scale with extremes from always to never |

**Additional file** 2: Indicators included in questionnaire pre-test and post-test (Continued)

| **Personal indicators** | | | | |
| --- | --- | --- | --- | --- |
| **Sense of coherence** | Life Orientation Questionnaire [9, 10] | Scale consist of 3 domains: meaningfulness, manageability and comprehensibility  Questions concern several aspects of life | 13 | 7-point scale with extremes from fully agree to fully disagree |
| **Life events** | Life events [11, 12] | Appearance of life events in past 12 months | 12 | Yes or no |
