# Supplementary material for: Evaluation design for a complex intervention program targeting loneliness in non-institutionalized elderly Dutch people
Source: BMC Public Health. 2010 Sep 13;10:552. doi: 10.1186/1471-2458-10-552 (PMC2945949; doi:10.1186/1471-2458-10-552)
Supplement: Additional file 3 — Indicators and methods to assess inputs, activities, outputs, and outcomes within Healthy Ageing. overview of the research activities in the process and effect evaluation of Healthy Ageing. Research activities are ordered along the components of the Logic Model for Loneliness Prevention (input, activities, outputs, short-term outcomes, mid-term outcomes, and long-term outcomes). Per intervention component the indicators and data collection methods are given. [file 1471-2458-10-552-S3.DOC]

**Additional file 3: Indicators and methods to assess inputs, activities, outputs, and outcomes within *Healthy Ageing***

| **Component logic model** | **Intervention component** | **Indicators** | **Method** |
| --- | --- | --- | --- |
| **Input** | Reflection functioning project group | Appreciation of e.g. collaboration, contribution of different project members  Perceived value of e.g. project in general, ability to spend enough time and effort on the project, ability to contribute personal expertise to the project, agreement between members about goals, planning, and activities of project, mid-term successes of project  Opinion about e.g. expertise of project members, personal commitment, each organization’s interest in contributing to the project, working procedure within project group | Coordination Action Checklist* after one and two years; outcomes of checklist will be discussed within project group |
|  | Reflection functioning management group | Perceived aims of the project  Opinion about potential continuation of collaboration after project period | Short questionnaire by email after one and two years |
|  | All meetings of the project group and individual meetings with external stakeholders | Name of organization, topics discussed, decisions made | Registration by project members |
| **Activities** | All activities as mentioned in  Appendix 1 | Delivery: Number of press releases, published articles; number of distributed posters, flyers; number of organized courses, information meetings, workshops, activities of *Neighbors Connected*, other social activities  Duration of an activity (once-off or repeated meetings); length of meetings; interval between meetings | Minutes of meetings  Registration by project members |

* Wagemakers A, Koelen M, Lezwijn J, Vaandrager L: Coordinated Action Checklist, a tool for partnerships to facilitate and evaluate community health promotion. *^Global Health Promotion^*^, in press^.

**Additional file 3: Indicators and methods to assess inputs, activities, outputs, and outcomes within *Healthy Ageing* (Continued)**

| **Outputs** | Information meetings | Appreciation of hosting organization about meeting in general, discussed topics, length of meeting, cooperation between presenters, information material  Appreciation of participants about meeting in general Advices to improve the meeting  Intended behavioral change | Evaluation form for contact persons  Participants comment in visitors’ book after meeting  Observations and informal feedback by project members |
| --- | --- | --- | --- |
|  | Psychosocial course  ‘Look for a meaningful life’ | Appreciation of course in general, course leaders  Intention to recommend the course to other people with depressive complains | Standard evaluation forms of mental health service after course |
|  | Psychosocial course  ‘Life stories’ | Appreciation of course in general, course leaders, discussed topics, length of meetings, interval between meetings, time of course, group size, group ambience, fulfillment of expectations  Intention to recommend the course to other people with depressive complains | Standard evaluation forms of mental health service after course |
|  | Psychosocial course  ‘Living with a chronic disease’ | Appreciation of course in general, intake, organizational issues, course materials, discussed topics, examples used  Perceived usefulness of skills learned  Intention to recommend the course to other people with a chronic disease | Standard evaluation forms of mental health service after course |
|  | *Neighbors Connected* organizers | Appreciation of organized activity in general, support received from the coordinator  Insight into strategies used by organizers to invite participants | Observations by coordinator  Interviews with organizers |

**Additional file 3: Indicators and methods to assess inputs, activities, outputs, and outcomes within *Healthy Ageing* (Continued)**

| **Outputs** | *Neighbors Connected* participants | Appreciation of activity in general  Motivation to participate in the activity  Opinion about the way of being informed about or invited to the activity | Participants comment in visitors’ book after activity  Observations by coordinator  Interviews with participants |
| --- | --- | --- | --- |
|  | Workshop to recognize symptoms of loneliness | Fulfillment of expectations  Appreciation of topics discussed, course leaders, length of meeting | Evaluation form after meeting |
|  | Round table meetings | Appreciation of the meeting in general  Suggestions about how to improve the meeting | Participants comment in visitors’ book after meeting  Notes during interactive sessions |
|  | All activities target the elderly | Opinion on the communication about different intervention activities  Barriers and facilitators experienced in relation to use of intervention activities  Perceived outcome expectation for intervention activities | In-depth interviews among approximately 20 elderly persons from the intervention community |
|  | All activities targeted at the elderly (except posters and flyers) | Reach: number of participants on courses, meetings, workshops, activities of *Neighbors Connected*, other social activities; compliance of participants during courses  Estimation of age and gender distribution (observation)  Dose received: Participation in one or more intervention activities; elderly read or heard about the project. | Registration by course leaders/project members  Recall in post-test within intervention community |

**Additional file 3: Indicators and methods to assess inputs, activities, outputs, and outcomes within *Healthy Ageing* (Continued)**

| **Short-term outcomes** | Psychosocial course  ‘Look for a meaningful life’ | Perceived reduction of depressive symptoms since the start of the course  Perceived contribution of the course to the reduction of depressive symptoms  Perceived increase in feeling of control over personal life since the start of the course  Perceived contribution of the course to an increased feeling of control  Intention to ask for additional professional support if necessary | Standard evaluation forms of mental health service after course |
| --- | --- | --- | --- |
|  | Psychosocial course  ‘Life stories’ | Perceived contribution of the course to improved wellbeing  Perceived contribution of the course to increase insight into personal life experiences  Perceived value of reminisce of life stories to the experience of new inspiration for the future | Standard evaluation forms of mental health service after course |
|  | Psychosocial course  ‘Living with a chronic disease’ | Perceived contribution of the course to reduction of complains  Perceived contribution of the course to the experience of increased coping capacities  Perceived contribution of the course to reach personal goals  Intention to ask for additional professional support if a necessary | Standard evaluation forms of mental health service after course |

**Additional file 3: Indicators and methods to assess inputs, activities, outputs, and outcomes within *Healthy Ageing* (Continued)**

| **Short-term outcomes** | *Neighbors Connected* participants | Intention to participate in any social activity another time  Experienced changes in domains of Sense of Coherence (meaningfulness, manageability and comprehensibility) in daily life | Participants comment in visitors’ book after activity  Interviews |
| --- | --- | --- | --- |
|  | Workshop to recognize symptoms of loneliness | Perceived increase in knowledge about risk factors for loneliness  Perceived importance of being attentive to loneliness among the elderly  Perceived ability to recognize signs of loneliness  Perceived ability to help the elderly with feelings of loneliness, by accurate referral to other specialists | Evaluation form after meeting (and after project period) |
|  | Overall complex intervention | Knowledge, motivation, and abilities of the elderly with regard to social engagement and help searching behavior (‘loneliness health literacy’) | Post-test in intervention and control group |
| **Mid-term and long-term outcomes** | Overall complex intervention | Loneliness  Social participation  Network structure  Network function  (full description of indicators in Appendix 2) | Pre-test post-test in intervention and control group |
